# Children’s Autonomic Nervous System Reactivity Moderates the Relations between Family Adversity and Sleep Problems in Latino 5-Year Olds in the CHAMACOS Study

**DOI:** 10.3389/fpubh.2017.00155

**Published:** 2017-06-30

**Authors:** Abbey Alkon, W. Thomas Boyce, Torsten B. Neilands, Brenda Eskenazi

**Affiliations:** ^1^Department of Family Health Care Nursing, University of California, San Francisco, San Francisco, CA, United States; ^2^Center for Environmental Research and Children’s Health (CERCH), University of California, Berkeley, Berkeley, CA, United States; ^3^Division of Developmental Medicine, University of California, San Francisco, San Francisco, CA, United States; ^4^Center for AIDS Prevention Studies, University of California, San Francisco, San Francisco, CA, United States

**Keywords:** CHAMACOS, adversity, sleep, children, routines, life events

## Abstract

Sleep problems are common for young children especially if they live in adverse home environments. Some studies investigate if young children may also be at a higher risk of sleep problems if they have a specific biological sensitivity to adversity. This paper addresses the research question, does the relations between children’s exposure to family adversities and their sleep problems differ depending on their autonomic nervous system’s sensitivity to challenges? As part of a larger cohort study of Latino, low-income families, we assessed the cross-sectional relations among family demographics (education, marital status), adversities [routines, major life events (MLE)], and biological sensitivity as measured by autonomic nervous system (ANS) reactivity associated with parent-rated sleep problems when the children were 5 years old. Mothers were interviewed in English or Spanish and completed demographic, family, and child measures. The children completed a 15-min standardized protocol while continuous cardiac measures of the ANS [respiratory sinus arrhythmia (RSA), preejection period (PEP)] were collected during resting and four challenge conditions. Reactivity was defined as the mean of the responses to the four challenge conditions minus the first resting condition. Four ANS profiles, co-activation, co-inhibition, reciprocal low RSA and PEP reactivity, and reciprocal high RSA and PEP reactivity, were created by dichotomizing the reactivity scores as high or low reactivity. Logistic regression models showed there were significant main effects for children living in families with fewer daily routines having more sleep problems than for children living in families with daily routines. There were significant interactions for children with low PEP reactivity and for children with the reciprocal, low reactivity profiles who experienced major family life events in predicting children’s sleep problems. Children who had a reciprocal, low reactivity ANS profile had more sleep problems if they also experienced MLE than children who experienced fewer MLE. These findings suggest that children who experience family adversities have different risks for developing sleep problems depending on their biological sensitivity. Interventions are needed for young Latino children that support family routines and reduce the impact of family adversities to help them develop healthy sleep practices.

## Introduction

An estimated 20–25% of young children experience sleep problems, such as insufficient hours or low-quality sleep (1). A systematic review found that 2- to 5-year-old children slept an average of 11.5 to 12.0 h with a wide range from 9.1 to 14.2 h in a 24-h day (2) with boys sleeping fewer hours than girls (3). These findings suggest that some children are sleeping less than the 10–13 h recommended by the American Academy of Sleep Medicine (4). Young children who sleep an insufficient number of hours are at risk for having adverse health problems (5), self-regulation problems (6), and learning difficulties (7). Preschool-age children who had sleep routines had a lower prevalence of obesity (8) and high social–emotional health (9). Thus, the American Academy of Pediatrics (AAP) encourages parents to foster good sleep habits, also known as sleep hygiene (10), by establishing bedtime routines, regular bedtime and wake-up times, regular physical activity during the day, and a comfortable, quiet, dark bedroom (11). The AAP encourages parents to learn to recognize sleep problems (e.g., difficulty falling asleep, nighttime awakenings, loud or heavy breathing) so they can seek professional help to improve their child’s sleep hygiene (10).

There are multiple factors, including cultural, environmental, economic, and physiologic, that contribute to the high prevalence of sleep problems for preschool-age children. African-American and Latino preschool-age children have later bedtimes and fewer bedtime routines than White preschool-age children (12). Several studies showed that non-White (i.e., African-American, Latino) infants, children under 3 years of age (13), school-age children (3), and adolescents (14) sleep fewer hours at night than same-age White children. In a qualitative study of Latino parents with preschool-age children, parents had consistent bedtime routines but their other sleep hygiene practices were less than optimal (15). Latino children’s sleep hours were affected by their parents’ work and school schedules. In addition, children whose mothers had low education had less sleep efficiency (16), were less likely to have a regular bedtime (12), and slept fewer hours (13) than children whose parents had higher education.

Young children living in stressful home environments may lack optimal sleep environments that support the development of healthy sleep habits (17). For example, young children experiencing family adversity (18–21), such as marital conflicts (22, 23), mothers who are depressed (13, 24), or living in poverty (3, 12), have poor sleep quality, shorter sleep duration, and more sleep problems than children experiencing fewer adversities. Children living with single mothers had fewer bedtime routines than children living with both their mothers and biological fathers (12).

Several studies showed that children living in families that lacked family routines, such as a regular bedtime routine, had more sleep problems (12, 25). In a study of preschool-age children, those living in families with fewer families routines, measured using a chaos scale, were associated with bedtime resistance and total sleep problems (26). Preschool-age children who adhered to a bedtime routine had more nightly sleep minutes, as measured by actigraphy (27).

A few studies suggest that the reactivity of a child’s autonomic nervous system (ANS) may play a role in sleep disturbances. The ANS, comprised of two branches the parasympathetic nervous system (PNS) and the sympathetic nervous system (SNS), together maintain the body’s normal physiology, including cardiac function, digestion, and respirations (28). The PNS and SNS respond differently across different contexts and different individuals. The PNS serves a “rest and digest” function and it slows heart rate (HR) and promotes calmness and homeostasis under everyday circumstances (29). On the other hand, the SNS serves as the “fight or flight” response to stressful circumstances. PNS and SNS reactivity measures assess the individual’s physiologic response to emotional, physical, or cognitive challenges compared to a resting state (30).

Boyce and Ellis’ biological sensitivity to context (BSC) (31) theory identifies children’s individual differences in their physiologic sensitivities to life’s challenges. Highly reactive children are biologically sensitive to their environment; those living in adverse environments can have negative health and behavior problems but those living in nurturing environments can have positive outcomes (20, 32). When highly reactive children encounter stressful circumstances they experience ANS responsivity. Under nurturing living conditions, these children regulate their ANS and display adaptive strategies; conversely, under adverse conditions these children are vulnerable and may not be able to self-regulate and adapt to the difficult circumstances.

The Adaptive Calibration Model (ACM) expands on the BSC theory and describes four responsivity patterns, sensitive, buffered, vigilant, and unemotional, that develop under the joint influence of environmental exposures and genetic factors (33). Children with a sensitive responsivity pattern have a predominance of PNS exhibited as high PNS responsivity and moderate SNS responsivity. Sensitive children are high in inhibitory control, executive function, and delay of gratification (34). Children with a buffered responsivity pattern have more PNS activity than SNS activity and develop in environments with moderate stress. They are lower in anxiety and aggression and more sensitive to social feedback than children with vigilant and unemotional profiles. Children may develop vigilant responsivity patterns if they grow up in stressful environments; these children will display SNS dominance with high SNS and low PNS responsivity. There are sex differences in the behavioral outcomes for children with the vigilant pattern. In males, they have increased impulsivity, risk-taking, and aggressive behavior and in females; they are lower in impulsivity and risk-taking and display withdrawn behavior. Children with the unemotional responsivity patterns exhibit low PNS and SNS responsivity. These children are less sensitive to social feedback and increase their risk-taking behavior. Children with low resting HRs or low ANS reactivity have been shown to be at risk for delinquency and behavior problems later in life (35, 36).

Children’s PNS and SNS physiologic responses to laboratory challenges have been shown to indicate their ability to regulate or respond to family, peer, and school challenges (37, 38). Cardiac measures of the ANS include measures of the PNS using respiratory sinus arrhythmia (RSA) and SNS using preejection period (PEP). These ANS measures are assessed in the laboratory under resting and challenging conditions. RSA, an indirect measure of the PNS, is the periodic oscillation in sinus rhythm occurring at the frequency of respiration and manifested as an increase in HR with inspiration and a decrease during expiration. PEP is an indirect, non-invasive measure of the time measured in milliseconds between ventricular contraction and the opening of the aortic valve.

The two branches of the ANS have dynamic and complementary responses which have been conceptualized as a two-dimensional model of autonomic control (39). Several researchers combined the PNS and SNS reactivity into four ANS profiles, co-activation, co-inhibition, reciprocal PNS activation and SNS not activated (low reactivity), and reciprocal PNS withdrawal and sympathetic activation (classic reactivity) (30, 39). The distribution of ANS profiles differs by age and sample characteristics (20, 40, 41).

Studies show relations between the different ANS reactivity profiles and children’s behavior problems. Children with the co-activation profile had externalizing problems when exposed to marital conflict (42). Children with the co-inhibition profile had more negative emotional expressiveness (43) and externalizing behaviors (44) than children with other ANS profiles. Children with reciprocal, low reactivity had poor self-control and aggressive behaviors (18, 35, 45). Healthy children with the classic reactivity profiles had better emotion regulation than children with other ANS profiles (43) but in a study of children with sickle cell disease the children with the classic reactivity profile were at risk for behavior problems if they also experienced family stress (20).

A child’s ANS resting or reactivity measures can function as vulnerability or protective factor in contributing to or buffering sleep problems. For example, children with high resting RSA indices have been found to have fewer sleep problems than children with low resting RSA or more parasympathetic withdrawal (46). Conversely, in a cross-sectional study, school-age children with both low resting RSA and low RSA reactivity had poor sleep quality and shorter sleep duration (47). Similarly, in a study of school-age children, those with high RSA reactivity to a cognitive challenge had better sleep duration and sleep quality, according to both self-report and actigraphy, than children with low RSA reactivity (48).

Only a few studies of children’s sleep have assessed both PNS and SNS concurrently. In a study of mixed-income African-American and European-American school-age children, SNS (skin conductance) and PNS (cardiac RSA) reactivity was measured as the difference between a social evaluative stressor compared to a resting state (49). The children with a classic reactivity profile, both SNS activation and PNS withdrawal during the challenge compared to rest, had better sleep efficiency and longer sleep duration than children with the other ANS profiles. Conversely, a study of high income, ethnically homogenous school-age children in Finland found no relation between sleep quantity or quality and the children’s PNS or SNS reactivity, using the Trier Social Stress Test (50). Additionally, school-age children in Belgium who showed autonomic dysregulation (sympathetic/parasympathetic imbalance) during a resting state had low sleep quality, as measured by accelerometers (51). Similarly, preschool-age children in Japan with low resting heart rate variability, a measure used to approximate both the PNS and SNS, had shorter sleep nocturnal duration (<10 h) compared to children with longer sleep nocturnal duration (52). The studies of children’s PNS and SNS biological sensitivity and sleep problems showed inconsistent findings that may be attributed to different samples from different countries, different ANS measures, and different resting and challenge conditions.

The aim of this study is to examine both PNS and SNS reactivity alone and together as a moderator of the relations between family stressors [i.e., major life events (MLE)] and mother-reported sleep problems in 5-year old Latino children. The research questions addressed are: (1) Do children who experience many family adversities (i.e., family routines, MLE) have more sleep problems than children who experience fewer family adversities? We hypothesize that children who live in families with few family routines and frequent MLE will have more sleep problems. (2) Does ANS reactivity change the relations between children who experience frequent MLE and sleep problems? (2a) We hypothesize that children with low ANS reactivity (i.e., either PNS or SNS reactivity) and frequent MLE will have the most sleep problems. (2b) We hypothesize that children who have the ANS profile of both low PNS and low SNS reactivity and who experience frequent MLE will have the most sleep problems. We hypothesize that the models with ANS profiles will have stronger associations with sleep problems than models with one ANS branch, PNS or SNS reactivity.

## Materials and Methods

### Study Design and Sample

This study is part of a larger, ongoing birth cohort study entitled The Center for the Health Assessment of Mothers and Children of Salinas (CHAMACOS), which examines the relations of pesticides and other environmental exposures on the health of pregnant women and their children (53). Pregnant women were recruited from six prenatal clinics between October 1999 and October 2000. Eligible women were 18 years of age or older, less than 20 weeks gestation, Spanish or English speaking, eligible for California’s low-income health insurance program, Medi-Cal, and planning to deliver at the county hospital.

The University of California (UC), Berkeley’s and UC, San Francisco’s institutional review boards approved the study protocols and consent forms. Written informed consent was obtained from one of the child’s legal guardians.

Of the 601 women initially enrolled, 527 were followed through delivery of a singleton birth and followed at regular intervals. We included in the present study the 333 children followed to 5 years of age. Children were excluded if they had a medical condition that could affect their cardiac measures (*n* = 4), were not 5 years of age when the reactivity protocol was administered (*n* = 2), had incomplete reactivity data (*n* = 15), did not complete the reactivity protocol (*n* = 30), or reactivity scores were outliers (>3 SD) (*n* = 5). The final sample included 282 children who had complete ANS data.

### Data Collection Procedures

Demographic, child, and family data were gathered during interviews conducted in Spanish or English with the mothers by bilingual, bicultural interviewers. At the 5-year visit, children completed a 15-min reactivity protocol measuring the resting state and ANS responses to social, cognitive, physical, and emotional challenges (40). The protocol was administered by bilingual, bicultural staff in the child’s language of choice (Spanish or English) and conducted in private rooms in a research office (41).

### Family Measures

#### Family Routines

The Home Observation Measurement of the Environment—Short Form was completed at the 5-year visit and two items were used to create a family routines index (54). The family routines included two items on the frequency of family meals and reading with your child. The frequency of “family meals with the child” was categorized as at least daily (1) versus less than daily (0). The frequency of “reading with the child” was categorized as at least daily (1) versus less than daily (0). The family routines index summarizes the two items into a three-level categorical variable (0, 0.5, 1.0).

#### Family MLE

The family MLE was modified from the Coddington MLE scale (55) using 16 items of events that occurred in the last 18 months. The items included changes in family finances, parent had an emotional problem, close family member died, parent in jail, and close family member hospitalized. The Cronbach’s alpha for the 16-item MLE score was 0.43. Each item was rated as yes or no and the sum of the items created the MLE score. The MLE scores were normally distributed.

### Child Sleep Problem Measure

The 110-item Child Behavior Checklist was completed during the maternal interview and includes a 7-item sleep problem subscale (56). Sleep problems included the following items: does not want to sleep alone, trouble getting to sleep, sleeps less than most children during the day, nightmares, resists going to bed at night, wakes up often at night, and talks or cries out in sleep. Each item was rated as not true, somewhat or sometimes true, or very true or often. The items were summed to create a total score (range 0–14) and higher total scores reflect more sleep problems. The Cronbach’s alpha of the items in the sleep subscale was 0.65. Since the distribution of sleep problems was not normally distributed, the mean scores were dichotomized as few sleep problems with scores of two or less (0) and more sleep problems with scores of three or higher (1).

### Child ANS Measures

Children participated in a 13-min standardized protocol where impedance cardiography, electrocardiography (ECG), and respirations were continuously measured (40). During the resting conditions the children were read a calming story for 2 min before and after the challenges. There were four challenges administered in the same order by a trained psychometrician. (1) The 2-min social interview was based on a Gesell School Readiness Screening Test (57) and the psychometrician asked questions for 2 min about the child’s friends, favorite activities, and birthday. (2) The 2-min number recall challenge was based on the standardized test where the psychometrician asks the child to repeat sequences of numbers (58), (3) The 1-min physical challenge was a taste-identification challenge (59) in which the child was asked to identify two drops of concentrated lemon juice placed in the middle of the child’s tongue, and (4) The 2-min emotion-evoking video involved a fear-evoking video of boys walking on a railroad bridge when the train unexpectedly comes on the tracks preceded by a 2-min neutral video.

Four spot electrodes were placed on the neck and trunk to collect impedance and respiratory measures, and three spot electrodes were placed on the right clavicle, lower left rib, and right abdomen for ECG measures. Data were acquired using the Biopac MP150 and continuous ECG, Z_o_ (basal impedance), and d*Z*/d*t* (first derivative of the impedance signal) waveforms were recorded. A 4-milliamp AC current at 100 Hz was passed through the two current electrodes and Z_o_ and dZ/d*t* signals were acquired from the two voltage-recording electrodes. The Mindware software (www.mindwaretech.com) was used to score RSA and PEP.

Respiratory sinus arrhythmia scores were calculated using the interbeat intervals on the ECG waveform, respiratory rates derived from the impedance (e.g., d*Z*/d*t*) signal, and a bandwidth range of 0.15–0.80 Hz (60). As the parasympathetic influence on HR decreases, referred to as parasympathetic withdrawal, the RSA index decreases.

Preejection period is the time interval in milliseconds of the onset of ventricular depolarization (Q point on the ECG wave) and the onset of left ventricular ejection (B point on the d*Z*/d*t* wave) (61). As sympathetic activity increases, PEP shortens.

Autonomic nervous system data were filtered, extracted, and then scored using Mindware software. Minute-by-minute data cleaning procedures involved examining for artifact and a child’s data were deleted if more than 25% of the task minutes were unscorable. Cleaning procedures also included checking for outliers and minutes with greater than 3 SD from the sample mean; in this sample, there were no outliers. Five percent of the participants (*n* = 15) who completed the reactivity protocol had missing data due to child or parent refusals, equipment failure, or noisy data due to child movement or electrode displacement.

Consistent with other researchers (62), PEP and RSA reactivity scores were calculated as the mean response across the four challenge tasks minus the preceding 2-min resting episode. The ANS responses to the four challenge tasks were combined to create a mean score since there was not sufficient time to include a task-specific rest period between the challenges. High RSA reactivity (i.e., negative RSA difference score) indicates the child had parasympathetic withdrawal during the challenges compared to the resting state. High PEP reactivity (i.e., negative PEP difference score; PEP shortens during the challenges compared to rest) indicates the child had sympathetic activation during the challenges compared to the resting state. Low RSA reactivity (i.e., positive RSA difference score) indicates the child had parasympathetic activation (i.e., more vagal input) during the challenge compared to the resting state. Low PEP reactivity (i.e., positive PEP difference score; PEP lengthens during the challenges compared to rest) indicates the child had sympathetic inhibition during the challenge compared to the resting state. Lastly, RSA and PEP positive and negative reactivity scores were dichotomized as activation or withdrawal/inhibition and then categorized into four ANS profiles: co-activation, co-inhibition, reciprocal PNS activation/SNS not activated (low reactivity), or reciprocal SNS activation/PNS withdrawal (classic reactivity) (Table 1) (41). There were four children with no profile scores because one of their reactivity scores was zero.

**Table 1:** Definition of ANS profile scores and descriptive statistics, CHAMACOS, *n* = 278.

ANS profile	RSA reactivity	PEP reactivity	*n* (%)
Co-activation of PNS and SNS	+	−	54 (19.4)
Co-inhibition of PNS and SNS	−	+	77 (27.7)
Reciprocal PNS activation, SNS not activated	+	+	41 (14.8)
Reciprocal PNS withdrawal, SNS activation	−	−	106 (38.1)

*ANS, autonomic nervous system; PNS, parasympathetic nervous system; SNS, sympathetic nervous system; RSA, respiratory sinus arrhythmia; PEP, preejection period; +, positive reactivity score; −, negative reactivity score; ANS reactivity score, mean challenge minus resting condition*.

### Statistical Analysis

Analyses were conducted using Stata version 14.0 (StataCorp, College Station, TX, USA). Descriptive statistics were calculated for all demographic characteristics and variables in the logit models. Spearman or Pearson correlations were used to explore the relations between the independent (i.e., demographic covariates, routines, family life events, ANS reactivity) and dependent variables (i.e., sleep problems) included in the logit models. The following demographic covariates in the model were child’s age at the time of the ANS protocol, sex, maternal education (<high school or more education), and marital status (living with a partner or single). The models included an interaction term for ANS reactivity (dichotomous) × family MLEs (continuous). Six separate models were run with each of the interaction terms: RSA reactivity (positive versus negative reactivity), PEP reactivity (positive versus negative reactivity), and four ANS profiles (each ANS profile versus all the other profiles) [i.e., co-activation, co-inhibition, reciprocal PNS activation/SNS not activated (low reactivity), and reciprocal SNS activation/PNS withdrawal (classic reactivity) by family MLEs (Table 1)]. Statistically significant interactive effects were examined by computing simple slopes for the associations of MLEs at each level of ANS reactivity.

*A priori* levels of significance were set at *p* < 0.05.

## Results

The mothers were predominantly low-income, Spanish-speaking, Mexican-born, living with a partner, and had less than a high school education (Table 2). Twenty two percent of the families had daily routines of having meals together as a family and reading with their child (Table 2). The most frequent MLEs in the last 12 months were: child began a new school (58%), family finances changed (41%), close family member died (23%), close family member was hospitalized (20%), and brother or sister was born (20%) (Table 3). Twelve percent of the sample did not experience any major life event and 14% experienced five or more life events. There were no significant correlations between the independent and dependent variables with any of the demographic covariates. There were no significant or moderate correlations between the independent variables.

**Table 2:** Sample characteristics, CHAMACOS at 5 years (*N* = 282).

Demographic characteristic	No. (%)
**Child sex**	
Girls	144 (51)
Boys	138 (49)
**Language at home**	
Mostly Spanish	258 (91)
English and Spanish equally	12 (4)
English only	9 (3)
Other language, not Spanish or English	3 (1)
**Federal poverty level (FPL)**	
Living at 100% FPL	173 (62)
Living at 200% FPL	98 (35)
Living at or above 300% FPL	9 (3)
**Mother’s country of birth**	
Mexico	246 (87)
United States	33 (12)
Other country	3 (1)
**Years lived in US at time of child’s birth**	
≤5 years	122 (43)
Over 5 years	160 (57)
**Marital status**	
Living with a partner	249 (88)
Not living with a partner	33 (12)
**Mother’s education**	
Less than high school	226 (80)
High school or higher education	56 (20)
**Working status**	
Mother working	202 (72)
Mother not working	80 (28)
Father working	230 (92)
Father not working	19 (8)
**Family routines**	
Eats family meals together every day	196 (70)
Reads together every day	81 (29)
Eats family meals and reads together every day	61 (22)

**Table 3:** Independent and dependent variables, CHAMACOS (*N* = 282).

Independent variables	No. (%)	Mean (SD)	Range, *N*
**Major life events (MLE)**			
Child began new school	163 (58)		
Family finances changed	115 (41)		
Close family member died	65 (23)		
Close family member hospitalized	56 (20)		
Brother or sister was born	55 (20)		
Another adult moved in	51 (18)		
Parent got new job and had to move away	52 (18)		
Brother or sister left home	47 (16)		
Parent had emotional problem	34 (12)		
Parents separated or divorced	23 (8)		
Parent in jail	23 (8)		
Family started fighting more	15 (5)		
Child hospitalized	10 (4)		
Child has visible deformity	11 (4)		
Family member was a victim of a crime	6 (2)		
MLE		2.36 (1.7)	0 to 9, 280
Respiratory sinus arrhythmia reactivity		−0.18 (0.5)	−1.48 to 1.55, 281
Preejection period reactivity		-0.27 (1.6)	−5.01 to 4.82, 282
**Dependent variable**			
Sleep problems (somewhat true and very true)			
Does not want to sleep alone	201 (60)		
Resists going to bed at night	116 (35)		
Has nightmares	65 (20)		
Talks or cries out in sleep	67 (20)		
Has trouble getting to sleep	49 (15)		
Wakes up often at night	38 (11)		
Sleeps less than most children during day and night	27 (8)		
Sleep problems		2.01 (1.9)	0 to 12, 282

Twenty-six percent (*n* = 73) of the children had no sleep problems, 17% (*n* = 47) had one sleep problem, 24% (*n* = 68) had two sleep problems, and 33% (*n* = 93) had three or more sleep problems. The most common sleep problems were: does not want to sleep alone (60%); resists going to bed at night (35%); has nightmares (20%); and talks or cries out in sleep (20%) (Table 3).

As shown in Table 4 Model 1, children living in families with no daily eating and reading routines had significantly more sleep problems than children living in families with daily routines [Model 1: odds ratios (OR) 0.36; 95% confidence interval (CI): 0.16, 0.80]. There were no significant main effects for children who experienced frequent MLE, RSA reactivity, or PEP reactivity in association with sleep problems.

**Table 4:** Family major life events (MLE), autonomic nervous system (ANS) reactivity, and children’s sleep problems.

Independent variables (1 = reference group)	Adjusted odds ratios of sleep problems, 95% confidence intervals by ANS reactivity models		
	Model 1: main effects	Model 2: preejection period (PEP) reactivity × MLE	Model 3: reciprocal Profile [1 = low respiratory sinus arrhythmia (RSA) and low PEP reactivity] × MLE
Age at time of ANS	0.70 (0.41, 1.81)	0.87 (0.75, 1.01)	0.84 (0.72, 0.98)
Sex (female = 1)	0.87 (0.75, 1.00)	0.69 (0.41, 1.17)	0.70 (0.41, 1.20)
Maternal education (<high school = 1)	0.98 (0.51, 1.89)	0.86 (0.45, 1.65)	1.04 (0.53, 2.01)
Marital status (single = 1)	1.35 (0.70, 2.63)	1.30 (0.67, 2.53)	1.22 (0.62, 2.43)
Family routines	0.35 (0.16, 0.79)*	0.35 (0.16, 0.78)*	0.36 (0.16, 0.80)*
MLE	1.22 (0.95, 1.56)	1.37 (1.08, 1.76)*	1.04 (0.89, 1.22)
RSA reactivity	0.59 (0.34, 1.01)	–	–
PEP reactivity	1.07 (0.63, 1.83)	2.65 (0.98, 7.19)	–
Reciprocal profile	–	–	0.42 (0.09, 1.95)
MLE × PEP reactivity		0.71 (0.52, 0.97)*	
MLE × low PEP reactivity		1.37 (1.08,1.76)*	
MLE × not PEP reactivity		0.97 (0.80, 1.80)	
MLE × reciprocal			1.73 (1.05, 2.85)*
MLE × reciprocal low reactivity profile			1.80 (1.12, 2.90)*
MLEX not reciprocal low reactivity profile			1.04 (0.89, 1.22)
*n*	276	277	276

*p < 0.05.

**Figure 1:**
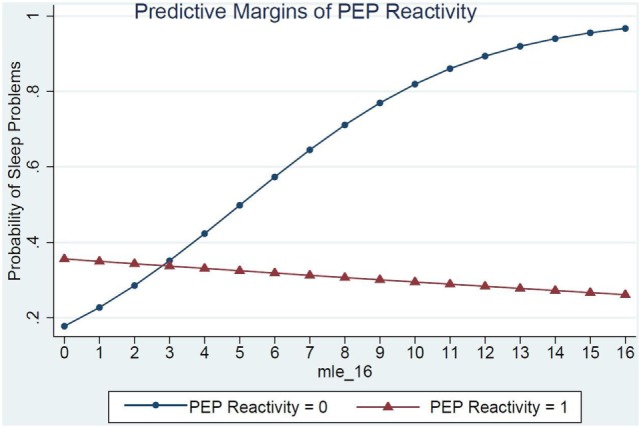
PEP reactivity as a moderator of the relations between major life events and sleep problems.

Children’s RSA reactivity did not modify the relations between MLEs and sleep problems (data not shown). However, when children’s PEP reactivity was in the model (Table 4, Model 2) it was a significant modifier of the relations between MLEs and sleep problems (*p* = 0.03) (Figure 1). For each additional MLE experienced by a child with low PEP reactivity, their odds of having a sleep problem increased by 37% (OR = 1.37, 95% CI: 1.08, 1.76). Children who had low PEP reactivity and experienced at least three MLEs had a steep increase in the probability of sleep problems for each additional MLE experienced. In contrast, children who did not have low PEP reactivity had no significant change in their sleep problems in relations to MLEs (OR = 0.97, 95% CI: 0.80, 1.80).

**Figure 2:**
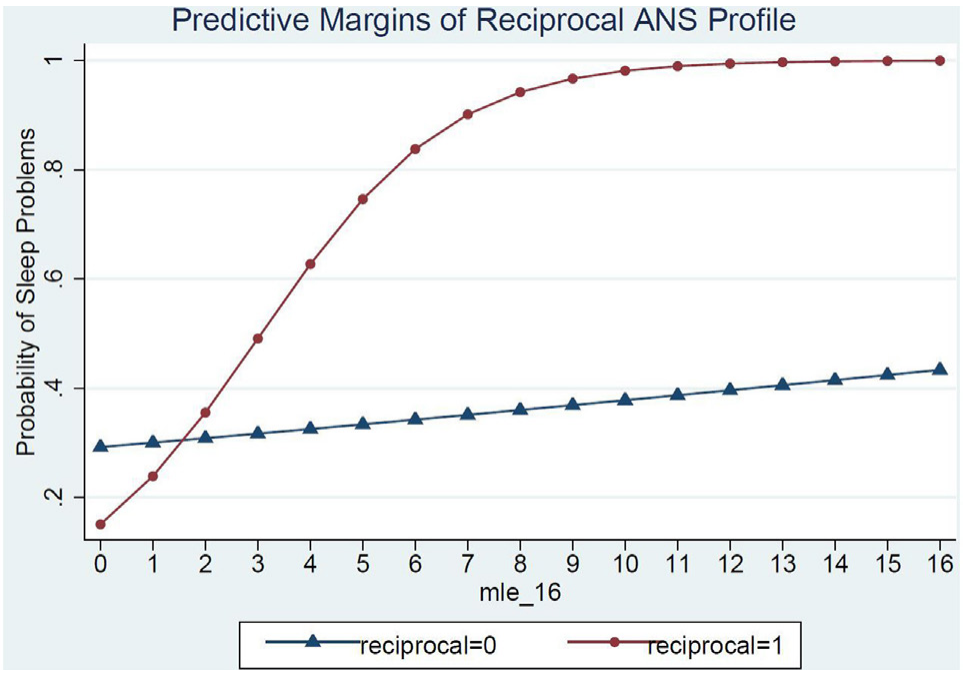
ANS low reactivity profile as a moderator of the relations between major life events and sleep problems.

Model 3 shows the ANS reactivity profile of reciprocal parasympathetic activation and not sympathetic activation (i.e., low PNS and SNS reactivity) as the modifier of the relations between MLE and sleep problems (Table 4). There was a significant interaction of ANS low reactivity profile and MLE and children’s sleep problems (*p* = 0.03) (Figure 2). Specifically, children with low PNS and RSA reactivity had higher odds of having sleep problems if they also experienced frequent MLEs compared to children with low PNS and RSA reactivity who experienced few MLEs. Simple slopes analysis of the interaction term revealed that for each additional MLE experienced by a child with the reciprocal, low reactivity profile their odds of having a sleep problem increased by 80% (OR = 1.80, 95% CI: 1.12, 2.90). Children who had low ANS reactivity profiles and experienced at least two MLEs had a steep increase in the probability of sleep problems for each additional MLE experienced. In contrast, children who did not have this low ANS reactivity profile had no significant change in their sleep problems in relations to MLEs (OR = 1.04, 95% CI: 0.89, 1.22).

Co-inhibition, co-activation, and classic reactivity ANS profiles did not significantly modify the relations of MLE on sleep problems.

## Discussion

This study of low-income, Latino preschool-age children examined the relations between adverse family events and children’s sleep problems and if these relations were modified by children’s ANS reactivity. Five-year old Latino children whose families had daily routines of eating family meals and reading together had fewer sleep problems than children whose families did not have these daily routines, after accounting for a child’s exposure to MLE and their biological sensitivity to laboratory stressors. Children who had low ANS reactivity profiles, including both the SNS and PNS, and also experienced multiple family adversities during the last 18 months had the highest probability of having sleep problems. Specifically, these children’s ANS reactivity was dominated by their low SNS, not PNS, reactivity. In summary, children who lived in families with few daily routines were at risk of having sleep problems and children who exhibited low biological sensitivity to challenges were at risk for sleep problems only under conditions of high family adversity.

The frequency of the sleep problems in this study is somewhat similar to other studies. In one study, 33% of the preschool-age children woke up at night at least once requiring their parents’ attention (63) compared to 11% of 5-year olds in the present study. In a convenience sample of 253 parents with young children, 23% of the children did not have a consistent bedtime and 56% frequently fell asleep with an adult present (64). In our study, parents reported that 60% of the children did not want to sleep alone.

Sleep problems identified during the preschool-ages tend to persist into school-age (65). In a 2-year longitudinal study of school-age children, those with sleep problems in the first year of the study were more likely to have sleep problems with decreased sleep quality and shorter sleep duration 1 year later compared to children without sleep problems at the start of the study (23).

There are subsequent health effects when children have short sleep duration and poor sleep quality. Insufficient sleep physiologically increases levels of ghrelin and decreases levels of leptin, which can affect appetite regulation and contribute to the development of obesity (5, 66, 67). There are many studies that show short sleep duration from 3 to 5 years of age is related to the development of obesity later in life (68, 69). Therefore, interventions to improve sleep duration and quality can have long term, positive health implications.

Our study showed that regular family bedtime routines may reduce the risk of sleep problems and thus, possibly mitigate the effects of adversity on sleep (25). In a national survey of 1,473 parents/caregivers of children from newborn to 10 years of age, a late bedtime and parental presence was associated with more night wakenings (63). For these preschool-age children, inconsistent bedtime routines and a television in the bedroom were associated with shorter total sleep duration. Thus, interventions on sleep hygiene and family routines can help improve young children’s sleep duration and quality.

Studies have shown that multiple, cumulative adversities experienced during early childhood have a stronger impact on children’s mental and physical health than single adversities (70–72). Our study’s findings also show that multiple adversities or MLE during early childhood have a negative impact on sleep problems for children with low ANS reactivity. Specifically, we found that three MLEs was the threshold for increasing the probability of sleep problems for children with low ANS reactivity.

Our sample’s RSA and PEP reactivity mean (SD) scores and low ANS reactivity profile prevalence are similar to another study of multi-ethnic 3- to 8-year olds (45% White) that used the same ANS protocol (40). The prevalence of ANS profiles is different in studies using other protocols or representing different populations. The prevalence of children with low RSA and PEP reactivity profiles was 5% for 4- to 9–year-old African-American children with sickle cell disease (73) and 2% for White and African-American school-age children and adolescents (30). Thus, the prevalence of ANS profiles may differ based on the sample’s ethnicity or race, age, and ANS protocol.

The polyvagal theory states that the PNS serves as a “brake” (i.e., blocks parasympathetic withdrawal) that decelerates HR under resting conditions. Under challenging conditions, the vagal brake can withdraw with a subsequent increase in arousal, but when this vagal mechanism is insufficient or dysregulated the SNS may be activated (74). If the SNS is activated and PNS withdraws (i.e., classic reactivity), there is a significant increase in HR. On the other hand, if there is low reactivity (PNS is activated and SNS is not activated), then HR decreases or remains slow. Under these circumstances, there may be little change from a resting to a challenge condition or the challenge condition may not activate the SNS or the PNS vagal brake.

Our findings support the ACM theory that children may develop an unemotional responsivity ANS profile as an adaptive response to severely stressful home environments (33). These extreme physiologic and neurobiologic responses to stressful experiences also stimulate or downregulate specific areas of the brain (75, 76). Under conditions of uncertainty and threat the prefrontal cortex becomes hypoactive and there is a subsequent increase in SNS responsivity. Prefontal hypoactivity has been associated with adult psychopathology, such as anxiety, depression, and post-traumatic stress disorder, and deficits in working memory and executive function (75). Our study included a sample of children experiencing many stressful experiences, with 97% of the children living in or near poverty and 88% of the children experiencing at least one major life event in the previous 18 months. Living with multiple adversities during early childhood may alter children’s ability to physiologically respond to environmental stressors and thus, some of these children develop a dampening or low ANS responsivity to challenging conditions. Chronic stress inhibits neurotransmitters and physically alters the structure and function of the limbic structures of the brain, hippocampus, amygdala, and prefrontal cortex, involved in controlling ANS responses to stress (77). In animal studies, chronic stress causes severe deficits in hippocampus-related memory and increases fear-motivated behavior (78, 79).

Several studies suggest that ANS reactivity may differ by type of challenge and context of the protocol (19, 80, 81). For example, school-age children with high reactivity to a cognitive, not interpersonal, challenge who experienced family conflict had the highest level of externalizing behavior problems (19). On the other hand, we found that the CHAMACOS children who experienced socioeconomic adversities in the first 5 years of life and had high reactivity to the social, not cognitive, challenge had high externalizing behavior problems at 7 years of age (82).

The stability of ANS reactivity has not been as strong as the stability of ANS resting measures (41, 60, 83). There is only one study of the CHAMACOS children from 6 months to 5 years of age that shows RSA and PEP resting measures are moderately stable but RSA and PEP reactivity is not stable. Since reactivity is not stable during these early years, it is hypothesized that this sensitive period of development is the time of plasticity for children to change their physiologic responses depending on their environment; thus, children’s reactivity may not stabilize until 6 years of age or older. This early period of development provides ample opportunity to offer interventions and support new parents to mitigate the negative effects of stress and adversity.

Several studies show direct relations between PNS activity and sleep problems. School-age children with PNS activation or less vagal withdrawal had more sleep problems and poorer sleep quality than children with greater vagal withdrawal during challenges (46). Children with high RSA reactivity had longer sleep duration and better sleep quality than children with low RSA reactivity (48). On the other hand, another study that found children with low RSA reactivity had lower sleep quality (47). Our findings did not support a relationship between RSA and sleep.

Although there are few studies of preschool-age children that include both RSA and PEP reactivity (52), there are several studies of older children (49–51). Children with reciprocal, low ANS reactivity show an overall dampened ANS responsivity during challenging conditions compared to a resting state which lowers their HR under challenging conditions (39). The reciprocal, low reactivity profile has been associated with children’s response to chronic stress described as allostatic load (74). Children who experience greater allostatic load tend to experience ANS hypo-reactivity when confronted with environmental stressors (28). Dampened SNS responsivity has been associated with low emotional expressiveness (43), antisocial behavior problems (84), and externalizing behavior problems (18, 85). A study of middle school children that found ANS dampening, reciprocal ANS activation, interfered with optimal sleep (49). Children with low reactivity may also have low arousal or be disengaged during the ANS protocol challenges.

A few studies supported our findings that children’s ANS reactivity moderated the relations between exposure to family adversities and sleep problems, but these studies showed positive relations for RSA. Children who experienced interparental conflict as stressful and had low RSA reactivity had more sleep problems with shorter sleep duration and decreased sleep quality (23). In another study, children who were exposed to maternal depression and had low RSA reactivity had a decrease in physical activity during sleep (24). In other studies of young children with low RSA reactivity, they had more distress and expression of negative emotions (86) and worse social skills and more externalizing behavior problems compared to same-age children with high RSA reactivity (87). There are no known studies of ANS profiles or PEP reactivity moderating relations between family adversities and sleep problems.

### Limitations

Although this study reports some novel findings there are several limitations. This cross-sectional design did not include longitudinal measures of family routines, MLE, and ANS reactivity, therefore it is not known if these factors predict sleep problems later in life. The parent-report measure of sleep problems is not objective and thus, our findings may not be comparable to studies that used actigraphy as the measure of sleep efficiency, quality, and duration. The adversity measures were limited and did not include potential confounders, such as the impact of neighborhoods, parent psychopathology, parental involvement and warmth, and cultural orientation or ethnic identity on children’s sleep. The majority of children in this sample were exposed to socioeconomic adversities, such as poverty and low maternal education, so there was a lack of variability in the sample’s demographics which may underestimate our findings. We summarized children’s reactivity responses as four challenges, rather than separating reactivity by challenge, and thus we cannot differentiate cognitive versus physical reactivity in relations to sleep problems. Finally, the findings from this study cannot be generalized beyond low-income, Mexican American populations living in the US.

### Implications and Future Studies

The implications of these novel findings are that interventions are needed to move from science to practice. Interventions for Latino families should include the importance of daily family routines as an approach to improving young children’s sleep. In addition, targeted interventions can identify children’s biological sensitivity to their environment as a measure of their individual differences to help the most vulnerable children cope with family stressors experienced at home.

Future studies should include objective actigraphy to identify sleep duration, sleep efficiency, and sleep quality in addition to parent-reports of sleep problems and sleep hygiene. ANS measures should include challenge-specific control conditions to separate reactivity by context. Latino children need to be included in large-scale, multi-ethnic intervention trials to decrease the prevalence of sleep problems. These studies are needed to improve young children’s sleep hygiene, sleep duration, and sleep quality with the long term goal of reducing the prevalence of later health problems, such as obesity, learning, and mental health problems (88).

## Ethics Statement

This study was carried out in accordance with the recommendations of the UC, Berkeley’s and UC, San Francisco’s institutional review boards. All subjects gave written informed consent in accordance with the Declaration of Helsinki. The protocol was approved by the UC, Berkeley’s Institutional Review Board and UC, San Francisco’s Committee on Human Research.

## Author Contributions

AA was the lead author. AA developed the research questions, planned the design and data collection specific to adversity and ANS and supervised the data collection and scoring of the measures, analyzed the data, and wrote the manuscript. WB contributed to the conceptual and theoretical framework for the study along with the development of the ANS protocol. WB and the primary author collaborated on the data collection procedures, scoring, and analysis included in the manuscript. TN worked directly with the lead author on the analysis plan, reviewed the findings, and collaborated on the interpretation of the findings. BE is the principal investigator on the larger cohort study and oversees all aspects of the study design, instrument selection, data collection procedures, analysis, and interpretation of findings. WB, TN, and BE reviewed the manuscript.

## Conflict of Interest Statement

The authors declare that the research was conducted in the absence of any commercial or financial relationships that could be construed as a potential conflict of interest.

## References

[1] Mindell J Owens J Carskadon M Developmental features of sleep. Child Adolesc Psychiatr Clin N Am (1999) 8(4):695–725. 10553199

[2] Galland B Taylor B Elder D Herbison P. Normal sleep patterns in infants and children: a systematic review of observational studies. Sleep Med Rev (2012) 16:213–22. 10.1016/j.smrv.2011.06.001 21784676

[3] Biggs S Lushington K Martin A van den Heuvel C Kennedy J. Gender, socioeconomic, and ethnic differences in sleep patterns in school-aged children. Sleep Med (2013) 14:1304–9. 10.1016/j.sleep.2013.06.014 24074692

[4] Paruthi S Brooks LJ D’Ambrosio C Hall WA Kotagal S Lloyd RM Recommended amount of sleep for pediatric populations: a consensus statement of the American Academy of Sleep Medicine. J Clin Sleep Med (2016) 12(6):785–6. 10.5664/jcsm.5866 27250809 PMC4877308

[5] Lumeng JC Somashekar D Appugliese D Kaciroti N Corwyn RF Bradley RH. Shorter sleep duration is associated with increased risk for being overweight at ages 9 to 12 years. Pediatrics (2007) 120(5):1020–9. 10.1542/peds.2006-3295 17974739

[6] Williams K Sciberras E. Sleep and self-regulation from birth to 7 years: a retrospective study of children with and without attention-deficit hyperactivity disorder at 8 to 9 years. J Dev Behav Pediatr (2016) 37(5):385–94. 10.1097/DBP.0000000000000281 26982247

[7] Gomez R Edgin J. Sleep as a window into early neural development: shifts in sleep-dependent learning effects across early childhood. Child Dev Perspect (2015) 9(3):183–9. 10.1111/cdep.12130 26557155 PMC4636128

[8] Anderson S Whitaker R. Household routines and obesity in US preschool-aged children. Pediatrics (2010) 125(3):420–8. 10.1542/peds.2009-0417 20142280

[9] Muniz E Silver E Stein R Family routines and social-emotional school readiness among preschool-age children. J Dev Behav Pediatr (2014) 35(2):93–9. 10.1097/DBP.0000000000000021 24509054

[10] American Academy of Pediatrics. Healthy Sleep Habits: How Many Hours Does Your Child Need? Elk Grove Village, IL: American Academy of Pediatrics (2016). Available from: https://healthychildren.org

[11] Owens J Witmans M Sleep problems. Curr Probl Pediatr Adolesc Health Care (2004) 34(4):154–79. 10.1016/j.cppeds.2003.10.003 15060483

[12] Hale L Berger L LeBourgeois M Brooks-Gunn J. Social and demographic predictors of preschoolers’ bedtime routines. J Dev Behav Pediatr (2009) 30(5):394–402. 10.1097/DBP.0b013e3181ba0e64 19745760 PMC2793084

[13] Nevarex M Rifas-Shiman S Kleinman K Gillman M Taveras E Associations of early life risk factors with infant sleep duration. Acad Pediatr (2010) 10(3):187–93. 10.1016/j.acap.2010.01.007 20347414 PMC2866807

[14] Matthews K Hall M Dahl R. Sleep in healthy black and white adolescents. Pediatrics (2014) 133(5):e1189–96. 10.1542/peds.2013-2399 24753532 PMC4006433

[15] Martinez S Thompson-Lastad A. Latino parents’ insight on optimal sleep for their preschool-age child: does context matter? Acad Pediatr (2015) 15(6):636–43. 10.1016/j.acap.2015.07.003 26547544

[16] El-Sheikh M Bagley EJ Keiley M Elmore-Staton L Chen E Buckhalth JA Economic adversity and children’s sleep problems: multiple indicators and moderation of effects. Health Psychol (2013) 32(8):849–59. 10.1037/a0030413 23148451 PMC4008970

[17] Dahl R The development and disorders of sleep. Adv Pediatr (1998) 45:73–90. 9742298

[18] Boyce WT Quas J Alkon A Smider NA Essex MJ Kupfer DJ Autonomic reactivity and psychopathology in middle childhood. Br J Psychiatry (2001) 179:144–50. 10.1192/bjp.179.2.144 11483476

[19] Obradovic J Bush N Boyce W. The interactive effect of marital conflict and stress reactivity on externalizing and internalizing symptoms: the role of laboratory stressors. Dev Psychopathol (2011) 23:101–14. 10.1017/S0954579410000672 21262042

[20] Treadwell M Alkon A Quirolo K Boyce W. Stress reactivity as a moderator of family stress, physical and mental health, and functional impairment for children with sickle cell disease. J Dev Behav Pediatr (2010) 31(6):491–7. 10.1097/DBP.0b013e3181e2830f 20585265 PMC4593493

[21] El-Sheikh M Harger J Whitson S. Exposure to interparental conflict and children’s adjustment and physical health: the moderating role of vagal tone. Child Dev (2001) 72(6):1617–36. 10.1111/1467-8624.00369 11768136

[22] El-Sheikh M Buckhalt J Cummings E Keller P. Sleep disruptions and emotional insecurity are pathways of risk for children. J Child Psychol Psychiatry (2007) 48(1):88–96. 10.1111/j.1469-7610.2006.01604.x 17244274

[23] El-Sheikh M Hinnant J Erath S Marital conflict, vagal regulation, and children’s sleep: a longitudinal investigation. In: El-Sheikh M Sadeh A, editors. Sleep and Development: Advancing Theory and Research. (Vol. 80), Wiley, Boston, MA: Monographs of the Society of Research in Child Development (2015). p. 89–106. 10.1111/mono.12146 25704737

[24] Keller P Kouros C Erath S Dahl R El-Sheikh M Longitudinal relations between maternal depressive symptoms and child sleep problems: the role of parasympathetic nervous system reactivity. J Child Psychol Psychiatry (2014) 55(2):172–9. 10.1111/jcpp.12151 24117807 PMC3947101

[25] Mindell J Li A Sadeh A Kwon R Goh D. Bedtime routines for young children: a dose-dependent association with sleep outcomes. Sleep (2015) 38(5):717–22. 10.5665/sleep.4662 25325483 PMC4402657

[26] Boles RE Halbower AC Daniels S Gunnarsdottir T Whitesell N Johnson SL. Family chaos and child functioning in relation to sleep problems among children at risk for obesity. Behav Sleep Med (2017) 15:114–28. 10.1080/15402002.2015.1104687 26745822 PMC4938783

[27] Staples A Bates J Petersen I Bedtime routines in early childhood: prevalence, consistency, and associations with nighttime sleep. In: El-Sheikh M Sadeh A, editors. Sleep and Development: Advancing Theory and Research. (Vol. 80), Wiley, Boston, MA: Monographs of the Society of Research in Child Development (2015). p. 141–59. 10.1111/mono.12149 PMC4843998 25704740

[28] Alkon A Wolff B Boyce W Chapter 12: Poverty, stress, and autonomic reactivity. In: Maholmes V King R, editors. The Oxford Handbook of Poverty and Child Development. New York: Oxford University Press (2012). p. 221–39.

[29] Porges S. The polyvagal perspective. Biol Psychol (2007) 74(2):116–43. 10.1016/j.biopsycho.2006.06.009 17049418 PMC1868418

[30] Salomon K Matthews K Allen M. Patterns of sympathetic and parasympathetic reactivity in a sample of children and adolescents. Psychophysiology (2000) 37:842–9. 10.1111/1469-8986.3760842 11117464

[31] Boyce W Ellis B. Biological sensitivity to context: I. An evolutionary-developmental theory of the origins and functions of stress reactivity. Dev Psychopathol (2005) 17(2):271–301. 10.1017/S0954579405050145 16761546

[32] Boyce WT Chesney M Alkon A Tschann JM Adams S Chesterman B Psychobiologic reactivity to stress and childhood respiratory illnesses: results of two prospective studies. Psychosom Med (1995) 57:411–22. 10.1097/00006842-199509000-00001 8552730

[33] Del Giudice M Ellis B Shirtcliff E. The adaptive calibration model of stress responsivity. Neurosci Biobehav Rev (2011) 35(7):1562–92. 10.1016/j.neubiorev.2010.11.007 21145350 PMC3068241

[34] Ellis B Boyce W Biological sensitivity to context. Curr Dir Pyschol Sci (2008) 17(3):183–7. 10.1111/j.1467-8721.2008.00571.x

[35] Raine A Autonomic nervous system factors underlying disinhibited, antisocial, and violent behavior. In: Ferris C Grisso T, editors. Understanding Aggressive Behavior in Children. (Vol. 794), New York: Annals of the NY Academy of Sciences (1996). p. 46–59. 10.1111/j.1749-6632.1996.tb32508.x 8853591

[36] Raine A Venables P Williams M. Relationships between central and autonomic measures of arousal at age 15 years and criminality at age 24 years. Arch Gen Psychiatry (1990) 47:1003–7. 10.1001/archpsyc.1990.01810230019003 2241502

[37] Obradovic J Bush N Stamperdahl J Adler N Boyce W. Biological sensitivity to context: the interactive effects of stress reactivity and family adversity on socioemotional behavior and school readiness. Child Dev (2010) 81(1):270–89. 10.1111/j.1467-8624.2009.01394.x 20331667 PMC2846098

[38] Essex M Armstrong J Burk L Goldsmith H Boyce W. Biological sensitivity to context moderates the effects of the early teacher-child relationship on the development of mental health by adolescence. Dev Psychopathol (2011) 23:149–61. 10.1017/S0954579410000702 21262045 PMC3058902

[39] Berntson G Cacioppo J Quigley K. Autonomic determinism: the modes of autonomic control, the doctrine of autonomic space, and the laws of autonomic constraint. Psychol Rev (1991) 98(4):459–87. 10.1037/0033-295X.98.4.459 1660159

[40] Alkon A Goldstein LH Smider N Essex MJ Kupfer DJ Boyce WT. Developmental and contextual influences on autonomic reactivity in young children. Dev Psychobiol (2003) 42:64–78. 10.1002/dev.10082 12471637

[41] Alkon A Boyce W Davis N Eskenazi B Developmental changes in autonomic nervous system resting and reactivity states in Latino children six to sixty months of age. J Dev Behav Pediatr (2011) 32:668–77. 10.1097/DBP.0b013e3182331fa6 22008788

[42] El-Sheikh M Kouros CD Erath S Cummings EM Keller P Staton L. Marital conflict and children’s externalizing behavior: interactions between parasympathetic and sympathetic nervous system activity. Monogr Soc Res Child Dev (2009) 74(1):1–97. 10.1111/j.1540-5834.2009.00501.x 19302676 PMC2918238

[43] Stifter C Dollar J Cipriano E. Temperament and emotion regulation: the role of autonomic nervous system reactivity. Dev Psychobiol (2011) 53:266–79. 10.1002/dev.20519 21400489 PMC3737744

[44] Beauchaine TP Gatzke-Kopp L Neuhaus E Chipman J Reid MJ Webster-Stratton C. Sympathetic- and parasympathetic-linked cardiac function and prediction of externalizing behavior, emotion regulation, and prosocial behavior among preschoolers treated for ADHD. J Consult Clin Psychol (2013) 81(3):481–93. 10.1037/a0032302 23544677 PMC3952490

[45] Ishikawa S Raine A Lencz T Bihrle S Lacasse L. Autonomic stress reactivity and executive functions in successful and unsuccessful criminal psychopaths from the community. J Abnorm Child Psychol (2001) 110(3):423–32. 10.1037/0021-843X.110.3.423 11502085

[46] Elmore-Staton L El-Skeikh M Vaughn B Arsiwalla D Preschoolers’ daytime respiratory sinus arrythmia and nighttime sleep. Physiol Behav (2012) 107:414–7. 10.1016/j.physbeh.2012.07.005 22842009

[47] El-Sheikh M Erath S Bagley E. Parasympathetic nervous system activity and children’s sleep. J Sleep Res (2013) 22:282–8. 10.1111/jsr.12019 23217056 PMC3912748

[48] El-Sheikh M Buckhalt J. Vagal regulation and emotional intensity predict children’s sleep problems. Dev Psychobiol (2005) 46:307–17. 10.1002/dev.20066 15832322

[49] Erath S El-Sheikh M Linking bioregulatory systems: reciprocal autonomic activation predicts sleep over 1 year in middle childhood. Dev Psychobiol (2015) 57(1):17–24. 10.1002/dev.21246 25230991

[50] Martikainen S Pesonen AK Feldt K Jones A Lahti J Pyhälä R Poor sleep and cardiovascular function in children. Hypertension (2011) 58:16–21. 10.1161/HYPERTENSIONAHA.111.172395 21555678

[51] Michels N Clays E De Buyzere M Vanaelst B De Henauw S Sioen I. Children’s sleep and autonomic function: low sleep quality has an impact on heart rate variability. Sleep (2013) 36(12):1939–46. 10.5665/sleep.3234 24293769 PMC3825444

[52] Sampei M Murata K Dakeishi M Wood D. Cardiac autonomic hypofunction in preschool children with short nocturnal sleep. Tohoku J Exp Med (2006) 208:235–42. 10.1620/tjem.208.235 16498231

[53] Eskenazi B Bradman A Gladstone EA Jaramillo S Birch K Holland N CHAMACOS, a longitudinal birth cohort study: lessons from the fields. J Child Health (2003) 1(1):3–27. 10.3109/713610244

[54] Caldwell B Bradley R Home Observation for Measurement of the Environment. Little Rock, AK: University at Arkansas at Little Rock (1984).

[55] Coddington R The significance of life events as etiologic factors in the diseases of children – II: a study of a normal population. J Psychosom Res (1972) 16:205–13. 10.1016/0022-3999(72)90018-9 5072914

[56] Achenbach T Manual for the Child Behavior Checklist/4-18 and 1991 Profile. Burlington, VT: University of Vermont, Department of Psychiatry (1991).

[57] Carlson R Gesell school readiness test. In: Keyser D Sweetland R, editors. Test Critiques. Kansas City: Test Corp of America (1985).

[58] Kaufman A Kaufman N Kaufman Assessment Battery for Children. Circle Pines, MN: American Guidance Service (1983).

[59] Kagan J Snidman N. Temperamental factors in human development. Am Psychol (1991) 46(8):856–62. 10.1037/0003-066X.46.8.856 1928938

[60] Bar-Haim Y Marshall P Fox N. Developmental changes in heart period and high-frequency heart period variability from 4 months to 4 years of age. Dev Psychobiol (2000) 37:44–56. 10.1002/1098-2302(200007)37:1<44::AID-DEV6>3.0.CO;2-7 10937660

[61] Kelsey R Guethlein W. An evaluation of the ensemble averaged impedance cardiogram. Psychophysiology (1990) 27(1):24–33. 10.1111/j.1469-8986.1990.tb02173.x 2339185

[62] Allen M Matthews K. Hemodynamic responses to laboratory stressors in children and adolescents: the influences of age, race, and gender. Psychophysiology (1997) 34:329–39. 10.1111/j.1469-8986.1997.tb02403.x 9175447

[63] Mindell J Meltzer L Carskadon M Chervin R. Developmental aspects of sleep hygiene: findings from the 2004 National Sleep Foundation Sleep in America Poll. Sleep Med (2009) 10:771–9. 10.1016/j.sleep.2008.07.016 19285450

[64] Owens J Jones C Nash R. Caregivers’ knowledge, behavior, and attitudes regarding healthy sleep in young children. J Clin Sleep Med (2011) 7(4):345–50. 10.5664/JCSM.1186 21897770 PMC3161766

[65] Simard V Nielsen T Tremblay R Boivin M Montplaisir J. Longitudinal study of preschool sleep disturbance: the predictive role of maladaptive parental behaviors, early sleep problems, and child/mother psychological factors. Arch Pediatr Adolesc Med (2008) 162(4):360–7. 10.1001/archpedi.162.4.360 18391145

[66] Snell E Adam E Duncan G Sleep and body mass index and overweight status of children and adolescents. Child Dev (2007) 78(1):309–23. 10.1111/j.1467-8624.2007.00999.x 17328707

[67] Hart CN Carskadon MA Considine RV Fava JL Lawton J Raynor HA Changes in children’s sleep duration on food intake, weight, and leptin. Pediatrics (2013) 132(6):e1473–80. 10.1542/peds.2013-1274 24190680

[68] Suglia S Duarte C Chambers E Boynton-Jarrett R. Social and behavioral risk factors for obesity in early childhood. J Dev Behav Pediatr (2013) 34(8):549–56. 10.1097/DBP.0b013e3182a509c0 24131877 PMC3960979

[69] Agras W Hammer L McNicholas F Kraemer H. Risk factors for childhood overweight: a prospective study from birth to 9.5 years. J Pediatr (2004) 145:20–5. 10.1016/j.jpeds.2004.03.023 15238901

[70] Sameroff A Seifer R Zax M Barocas R. Early indicators of developmental risk: Rochester Longitudinal Study. Schizophr Bull (1987) 13(3):383–94. 10.1093/schbul/13.3.383 3629195

[71] Evans G English K. The environment of poverty: multiple stressor exposure, psychophysiological stress, and socioemotional adjustment. Child Dev (2002) 73(4):1238–48. 10.1111/1467-8624.00469 12146745

[72] Chartier M Walker J Naimark B. Separate and cumulative effects of adverse childhood experiences in predicting adult health and health care utilization. Child Abuse Negl (2010) 34:454–64. 10.1016/j.chiabu.2009.09.020 20409586

[73] Pearson SR Alkon A Treadwell M Wolff B Quirolo K Boyce WT Autonomic reactivity and clinical severity in children with sickle cell disease. Clin Auton Res (2005) 15:400–7. 10.1007/s10286-005-0300-9 16362543

[74] McEwen BS Protective and damaging effects of stress mediators. N Engl J Med (1998) 338(3):171–9. 10.1056/NEJM199801153380307 9428819

[75] Thayer JF RD L Claude Bernard and the heart–brain connection: further elaboration of a model of neurovisceral integration. Neurosci Biobehav Rev (2009) 33:81–8. 10.1016/j.neubiorev.2008.08.004 18771686

[76] McEwen BS. Physiology and neurobiology of stress and adaptation: central role of the brain. Physiol Rev (2007) 87:873–904. 10.1152/physrev.00041.2006 17615391

[77] Ulrich-Lai Y Herman J. Neural regulation of endocrine and autonomic stress responses. Nat Rev Neurosci (2009) 10:397–409. 10.1038/nrn2647 19469025 PMC4240627

[78] de Kloet E Joels M Holsboer F. Stress and the brain: from adaptation to disease. Nat Rev Neurosci (2005) 6:463–75. 10.1038/nrn1683 15891777

[79] McKlveen JM Morano RL Fitzgerald M Zoubovsky S Cassella SN Scheimann JR Chronic stress increases prefrontal inhibition: a mechanism for stress-induced prefrontal dysfunction. Biol Psychol (2016) 80:754–64. 10.1016/j.biopsych.2016.03.2101 27241140 PMC5629635

[80] Obradovic J. How can the study of physiological reactivity contribute to our understanding of adversity and resilience processes in development? Dev Psychopathol (2012) 24:371–87. 10.1017/S0954579412000053 22559120

[81] Skowron E Cipriano-Essel E Gatzke-Kopp L Teti D Ammerman R Early adversity, RSA, and inhibitory control: evidence of children’s neurobiological sensitivity to social context. Dev Psychobiol (2014) 56:964–78. 10.1002/dev.21175 24142832 PMC3992193

[82] Alkon A Waters SF Boyce WT Johnson MM Harley KG Eskenazi B Latino children’s autonomic nervous system reactivity moderates the relations between cumulative socioeconomic adversity in the first five years and externalizing behavior problems at seven years. Adv Pediatr Res (2016) 3:6 10.12715/apr.2016.3.6

[83] De Rogalski Landrot I Roche F Pichot V Teyssier G Gaspoz JM Barthelemy JC Autonomic nervous system activity in premature and full-term infants from theoretical term to 7 years. Auton Neurosci Basic Clin (2007) 136:105–9. 10.1016/j.autneu.2007.04.008 17556047

[84] van Goozen S Fairchild G Snoek H Harold G The evidence of a neurobiological model of childhood antisocial behavior. Psychol Bull (2007) 133(1):149–82. 10.1037/0033-2909.133.1.149 17201574

[85] Beauchaine T Gatzke-Kopp L Mead H Polyvagal theory and developmental psychopathology: emotion dysregulation and conduct problems from preschool to adolescence. Biol Psychiatry (2007) 74:174–84. 10.1016/j.biopsycho.2005.08.008 PMC1801075 17045726

[86] Buss K Goldsmith H Davidson R. Cardiac reactivity is associated with changes in negative emotion in 24-month-olds. Dev Psychobiol (2005) 46:118–32. 10.1002/dev.20048 15732055

[87] Calkins S Keane S. Cardiac vagal regulation across the preschool period: stability, continuity, and implications for childhood adjustment. Dev Psychobiol (2004) 45:101–12. 10.1002/dev.20020 15505799

[88] Hale L Berger L Sleep duration and childhood obesity: moving from research to practice. Sleep (2011) 34(9):1153–4. 10.5665/SLEEP.1226 21886351 PMC3157655

